# Dual solution for double-diffusive mixed convection opposing flow through a vertical cylinder saturated in a Darcy porous media containing gyrotactic microorganisms

**DOI:** 10.1038/s41598-021-99277-x

**Published:** 2021-10-07

**Authors:** Abdulaziz Alsenafi, M. Ferdows

**Affiliations:** 1 Department of Mathematics, Kuwait University, Kuwait City, Kuwait; 2 Present Address: Research Group of Fluid Flow Modeling and Simulation, Department of Applied Mathematics, University of Dhaka, Dhaka, Bangladesh

**Keywords:** Engineering, Materials science, Mathematics and computing, Nanoscience and technology

## Abstract

The steady mixed convection flow towards an isothermal permeable vertical cylinder nested in a fluid-saturated porous medium is studied. The Darcy model is applied to observe bioconvection through porous media. The suspension of gyrotactic microorganisms is considered for various applications in bioconvection. Appropriate similarity variables are opted to attain the dimensionless form of governing equations. The resulting momentum, energy, concentration, and motile microorganism density equations are then solved numerically. The resulting dual solutions are graphically visualized and physically analyzed. The results indicate that depending on the systems' parameters, dual solutions exist in opposing flow beyond a critical point where both solutions are connected. Our results were also compared with existing literature.

## Introduction

The study of mixed convection, which is the combination of free and forced convection flow, has become of great interest for many researchers over the last few decades because of its wide range of technological and industrial applications that have been reviewed in Refs.^1–3^. This includes heat exchanges placed in a low velocity environment, solar collectors exposed to wind currents, atmospheric boundary layer flows, nuclear reactors when cooled during emergency shutdowns, and various electronic equipment. Convection heat transfer in porous medium has many theoretical and practical studies, such as in Refs.^4–7^, where the effects of buoyancy phenomena on flow and temperature fields through porous media were studied. In a porous medium, the pores are typically filled with fluid (liquid or gas), which causes enhance heat transfer in fluid flow.

Several studies were performed on convection heat sources that are based on the Darcy model^8–12^. For instance, in Ref.^8^, Lai et al. applied the Darcy model to observe mixed convection in porous media. Abbas et al.^9^ studied natural convection using the Darcy–Brinkman–Forcheimer model in a vertical cylinder. Srinivasacharya and Reddy^10,11^ studied the problem of natural and mixed convection for a power-law fluid in a Darcy porous media. In Ref.^12^, Naveen et al. studied the velocity term impacts of both the maximum density and the momentum equation on the stability of a natural convection through a vertical layer in a Darcy porous media. Furthermore, other studies were done on convection flow through porous media, such as those in Refs.^13–17^. Very recently, Mondal et al.^18^ observed internal heat generation and thermal radiation for mixed convection flow over a porous vertical plate. Mixed convection through porous media with heat generation is also studied by Abu-hamdeh et al.^19^ and Maleque^20^. Additionally, Shankar and Shivakumara studied the natural convection in a non-Newtonian Oldroyd-B fluid that is saturated in a vertical porous layer that is maintained at varying uniform temperatures^21–23^. By analyzing the systems’ stability, the authors found that the system is unconditionally stable for Newtonian fluids, and is unstable with viscoelastic fluids.

Flow over vertical cylinder has become of great interest to authors due to its numerous applications. For example, it is used to insulate vertical porous pipes, connect with oil/gas lines, underground electrical power transmission lines, radioactive waste disposal, polymer process, and heating or cooling of sheets and films. In Ref.^24^, Sankar and Do investigated the effects of discrete heating on convection heat transfer in a vertical cylindrical annulus. Several works have also been done on free convection heat transfer in vertical cylinder annulus^25–28^. Moreover, free convective boundary layer flows over a vertical porous cylinder have been investigated by Totala et al.^29^, Paul et al.^30^, Minkowyez and Cheng^31^. In Ref.^32^, Popiel observed free convection heat transfer from the vertical slender cylinder, and Loganathan et al.^33^ observed natural convection flow for a vertical moving cylinder. Several researchers, such as references^34–36^, studied mixed convection flow over a vertical cylinder. Very recently, Girish et al.^37^ studied mixed convection in vertical double annular passages through three coaxial cylinders, Rihan^38^ observed mixed convection over a short vertical cylinder, and Mkhatshwa et al.^39^ studied mixed convection nanofluid flow over a vertical slender cylinder.

Bioconvection can be classified as a development process in the field of fluid flow, which deals with the steps of self-propelled up swimming microorganisms, such as algae and bacteria that contain oxytaxis, gyrotaxis, and gravitaxis organisms. Motile microorganisms are heavier than their encompassing liquid and usually swim in the upward direction, which brings about producing different flow profiles into the system, as described briefly in Refs.^40–46^. The advantages of adding motile microorganisms to the suspension include improved mass transfer and microscale mixing. In Refs.^47,48^, Ghorai et al. observed the stability and development of gyrotactic microorganisms in an in-depth cavity. Mixed convection nanofluid flow containing gyrotactic microorganisms is observed by several researchers^49–51^. Moreover, Mahdy^49^ studied gyrotactic microorganism mixed convection flow along with isothermal vertical wedge. Khan et al.^50^ observed mixed convection in a gravity-driven thin film for non-Newtonian nanofluid with microorganisms, and Saleem et al.^51^ presented the behavior of magneto Jeffrey nanofluid with gyrotactic microorganisms over a rotating cone. Recently, Rashad et al.^52,53^ and Sudhagar et al.^54^ explored mixed convection nanofluid flow over a vertical circular cylinder containing gyrotactic microorganisms.

In convective heat transfer, there exist complex nonlinear problems. For highly nonlinear problems, multiple (dual) solutions can sometimes be obtained. It is important to compute unstable states along with stable ones as the unstable solutions often interact with stable solutions, which produce unexplainable phenomena, as observed by Rohni et al.^55^. The study on the existence of dual solutions in mixed convective boundary layer flows may bring a new outlook on engineering applications described in Ref.^56^. In Ref.^57^, Ridha et al. showed the existence of a dual solution for opposing flow. After that, Ref.^58^ extended on that research for assisting flow. Dual solutions for mixed convection boundary layer flow were first investigated by Ingham^59^. Merkin^60^ also studied dual solutions for mixed convection in a porous medium simultaneously. Ishak et al.^61^ and Rostami et al.^62^ investigated dual solutions on mixed convection over a vertical surface with micropolar fluid as described in Ref.^61^, as well as the presence of silica-alumina hybrid nanofluid in Ref.^62^. Very recently, Khan et al.^63^ also presented a dual solution for mixed convection with silica-alumina hybrid nanofluid for a curved surface.

Motivated by the above works, in this paper we present a dual solution for mixed convection over a vertical cylinder containing gyrotactic microorganisms in an opposing flow regime. Based on Refs.^50,52^, our work can be used in engineering, geothermal, and industrial domains, such as developing microbial fuel cells and bio-convection technological devices. Additionally, dual solutions mathematical analysis can determine the most realistic, stable, physically acceptable solutions that significantly impact designing those devices. Examples of engineering applications include but are not limited to power systems, where they are used in system planning and operation to predict the systems’ response. Even though several authors^3,36,64^ investigated dual solutions for mixed convection, they did not observe motile microorganism’s behavior in their studies. Moreover, according to our knowledge, very few works such as those mentioned in Refs.^52,65^ have been done on dual solutions for mixed convection with gyrotactic microorganisms, in which the behavior of nanofluid with microorganisms was studied. The novelty of this work is to observe dual solution phenomena in mixed convection opposing flow for water-based up-swimming microorganisms. This phenomenon has significant applicability potential in bio-microsystems. Analyzing the existence of a dual solution in heat, mass, motile microorganism transfer rate and temperature, concentration microorganism profile beyond a critical point along a vertical cylinder is completely a new concept, and the obtained results are entirely unique.

## Mathematical formulation

**Figure 1:**
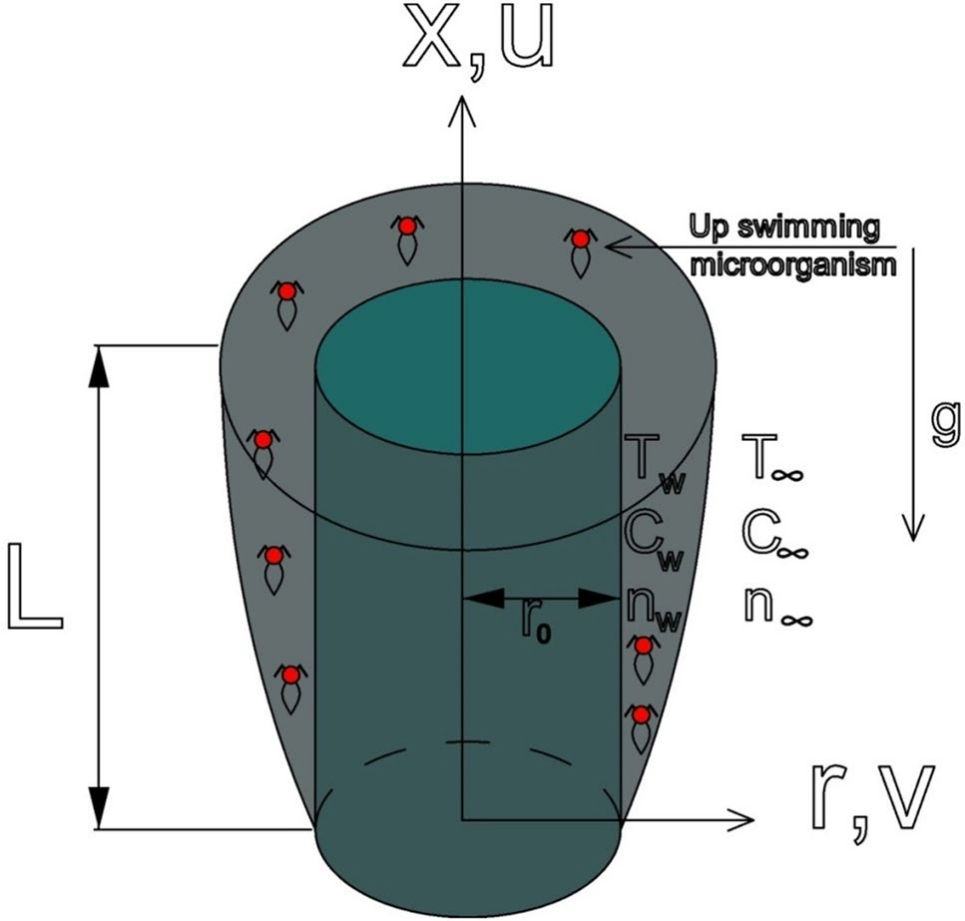
Physical model and coordinate system.

We consider the steady mixed convection boundary layer flow over a vertical cylinder with a radius ${r}_{0}$ implanted in a saturated permeable medium that contains gyrotactic microorganisms, as shown in Fig. 1. In our work, we assume that the mainstream velocity is $U\left(x\right)$ and the cylinder surface is maintained at a constant temperature of ${T}_{w}$. We denote the concentration of fluid by ${C}_{w}$ and motile microorganism concentration by ${n}_{w}$. The velocity, temperature, and concentrations are ${u}_{\infty }, {T}_{\infty }, {C}_{\infty }$ and ${n}_{\infty }$. When it is far from the cylinder’s surface, the axial and radial coordinates are $x$ and $r;$ in contrast, the $x$-axis is measured vertically upward along the cylinder’s axis, and the $r$-axis is measured normal to the $x$-axis. The gravitational acceleration $g$ acts in the downward direction in opposition to the $x$-direction.

We use the Darcy model in this research, and it assumes less velocity and porosity. It is worth mentioning that water has been chosen as the base fluid for the survival of microorganisms. The buoyancy term is used in the momentum (Darcy) equation due to up swimming microorganisms. Based on the model proposed by Sudnagar et al.^54^, under the assumptions along with the physical phenomena and Boussinesq approximations, the governing equations are

1 $$\begin{array}{c}\frac{\partial \left(ru\right)}{\partial x}+\frac{\partial \left(rv\right)}{\partial r}=0,\end{array}$$

2 $$\begin{array}{c}\frac{\partial u}{\partial r}=\frac{\left(1-{C}_{\infty }\right)\rho \beta gk}{\mu }\frac{\partial T}{\partial r}-\frac{\rho gk}{\mu }\frac{\partial C}{\partial r}-\frac{g\gamma \nabla \rho k}{\mu }\frac{\partial n}{\partial r},\end{array}$$

3 $$\begin{array}{c}u\frac{\partial T}{\partial x}+v\frac{\partial T}{\partial r}=\alpha \left(\frac{1}{r}\frac{\partial }{\partial r}\left(r\frac{\partial T}{\partial r}\right)\right),\end{array}$$

4 $$\begin{array}{c}u\frac{\partial C}{\partial x}+v\frac{\partial C}{\partial r}={D}_{m}\left(\frac{1}{r}\frac{\partial }{\partial r}\left(r\frac{\partial C}{\partial r}\right)\right),\end{array}$$

5 $$\begin{array}{c}u\frac{\partial n}{\partial x}+v\frac{\partial n}{\partial r}+\frac{b{W}_{c}}{\nabla C}\left(\frac{\partial }{\partial r}\left(n\frac{\partial C}{\partial r}\right)\right)={D}_{n}\left(\frac{1}{r}\frac{\partial }{\partial r}\left(r\frac{\partial n}{\partial r}\right)\right).\end{array}$$

In Eqs. (1) to (5) above, $T$, $C$, and $n$ are the temperature, concentration, and volume fraction of motile microorganisms. $k$ is the permeability of the porous medium, $\mu$ is the fluid viscosity, $\rho$ is the density of the fluid, $g$ is the acceleration due to gravity, β is the thermal expansion coefficient, $\alpha$ is the effective thermal diffusivity of the porous medium, ${D}_{m}$ is the solute diffusivity, ${D}_{n}$ is the diffusivity of the microorganism, $b$ is the chemotaxis constant, and finally, ${W}_{c}$ is the maximum cell swimming speed. The product ${b\cdot W}_{c}$ is assumed to be a constant.

The boundary conditions take the following form:

6 $$\begin{array}{c}v=0, T={T}_{w}\left(x\right), C={C}_{w}\left(x\right), n={n}_{w}\left(x\right) \, \, \, {\text{a}}{\text{t}} \, \, \, r={r}_{0},\end{array}$$

7 $$\begin{array}{c}u\to U\left(x\right), T\to {T}_{\infty }, C\to {C}_{\infty }, n\to {n}_{\infty }{\text{a}}{\text{s}} \, \, \, r\to \infty .\end{array}$$

Following Mahmood and Merkin^34^, we also assume in this paper the following:

8 $$\begin{array}{c}U\left(x\right)=\frac{{u}_{\infty }x}{L}, {T}_{w}\left(x\right)={T}_{\infty }+\frac{x\nabla T}{L}, {C}_{w}\left(x\right)={C}_{\infty }+\frac{x\nabla C}{L}, {n}_{w}\left(x\right)={n}_{\infty }+\frac{x\nabla n}{L}.\end{array}$$

We now introduce the following dimensionless quantities:

9 $$\begin{array}{c}\eta =\frac{{r}^{2}-{{r}_{0}}^{2}}{2{r}_{0}L}P{e}^\frac{1}{2}, \psi =\alpha {r}_{0}P{e}^\frac{1}{2}\frac{x}{L}f\left(\eta \right),\end{array}$$

10 $$\begin{array}{c}U\left(x\right)=\frac{\alpha xPe}{{L}^{2}}, T={T}_{\infty }+\frac{x\nabla T}{L}\theta \left(\eta \right), C={C}_{\infty }+\frac{x\nabla C}{L}\phi \left(\eta \right), n={n}_{\infty }+\frac{x\nabla n}{L} \chi \left(\eta \right),\end{array}$$

where $L$ is the characteristic length, and $Pe$ is the Peclet number.

The continuity equation is satisfied by a stream function $\psi$ such that:

$$u=\frac{1}{r}\frac{\partial \psi }{\partial r}\text{ and }v=-\frac{1}{r}\frac{\partial \psi }{\partial x}.$$

Substituting Eqs. (9) and (10) in Eqs. (1) to (7) leads to the following coupled differential equations:

11 $$\begin{array}{c}{f}^{^{\prime\prime} }=\lambda \left[{\theta }^{^{\prime}}-Nr{\phi }^{^{\prime}}-Rb{\chi }^{^{\prime}}\right],\end{array}$$

12 $$\begin{array}{c}\left(1+\gamma \eta \right){\theta }^{^{\prime\prime} }+\gamma {\theta }^{^{\prime}}+f{\theta }^{^{\prime}}-{f}^{^{\prime}}\theta =0,\end{array}$$

13 $$\begin{array}{c}\left(1+\gamma \eta \right){\phi }^{^{\prime\prime} }+\gamma {\phi }^{^{\prime}}+Le\cdot f{\phi }^{^{\prime}}-Le\cdot {f}^{^{\prime}}\phi =0,\end{array}$$

14 $$\begin{array}{c}\left(1+\gamma \eta \right){\chi }^{^{\prime\prime} }+\gamma {\chi }^{^{\prime}}+Lb\cdot f{\chi }^{^{\prime}}-Lb\cdot {f}^{^{\prime}}\chi -Pb\left(\left(1+\gamma \eta \right){\phi }^{^{\prime}}{\chi }^{^{\prime}}+\left(\chi +A\right)\left(\gamma {\phi }^{^{\prime}}+\left(1+\gamma \eta \right){\phi }^{^{\prime\prime} }\right)\right)=0.\end{array}$$

The transformed boundary conditions become:

15 $$\begin{array}{c}\eta =0, f=0, \theta =1, \phi =1, \chi =1,\end{array}$$

16 $$\begin{array}{c}\eta \to \infty , {f}^{^{\prime}}\to 1, \theta \to 0, \phi \to 0, \chi \to 0.\end{array}$$

In the coupled differential equations, the mixed convection parameter is $\lambda =\frac{Ra}{Pe}$, Raleigh number is $Ra=\frac{g\beta kL\nabla T}{\upsilon \alpha }$, Peclet number is $Pb=\frac{{u}_{\infty }L}{\alpha }$, curvature parameter is $\gamma =\frac{2}{{r}_{0}}\sqrt{\frac{\alpha L}{{u}_{\infty }}}$, buoyancy parameter is $Nr=\frac{\nabla C}{\left(1-{C}_{\infty }\right)\beta \nabla T}$, bioconvection Rayleigh number is $Rb=\frac{\nabla \rho \gamma \nabla n}{\left(1-{C}_{\infty }\right)\beta \nabla T}$, Lewis number is $Le=\frac{\alpha }{{D}_{m}}$, bioconvection Lewis number is $Lb=\frac{\alpha }{{D}_{n}}$, bioconvection Peclet number is $Pb=\frac{b{W}_{c}}{{D}_{n}}$, and the microorganism concentration difference parameter is $A=\frac{{n}_{\infty }}{{n}_{w}-{n}_{\infty }}$.

## Heat, mass, and motile microorganism transfer coefficient

The heat transfer rate, the Sherwood number, and the density parameter for the motile microorganisms are defined as:

17 $$\begin{array}{c}Nu=\frac{L{q}_{w}}{{k}_{eff}\nabla T}, Sh=\frac{L{q}_{m}}{{D}_{m}\nabla C}, and Nn=\frac{L{q}_{n}}{{D}_{n}\nabla n},\end{array}$$

where ${q}_{w}$, ${q}_{m}$, and ${q}_{n}$ represent the constant wall heat, mass, and microorganisms’ fluxes, respectively, and they are written as:

18 $$\begin{array}{c}{q}_{w}=-{k}_{eff}{\left(\frac{\partial T}{\partial r}\right)}_{r={r}_{0}}, {q}_{m}=-{D}_{m}{\left(\frac{\partial C}{\partial r}\right)}_{r={r}_{0}}, {q}_{n}=-{D}_{n}{\left(\frac{\partial n}{\partial r}\right)}_{r={r}_{0}}.\end{array}$$

By using Eqs. (9), (10), (17), and (18), we obtain the dimensionless Nusselt number, Sherwood number, and the local density number of the motile microorganisms at the surface of the cylinder, respectively:

19 $$\begin{array}{c}P{e}^{-\frac{1}{2}}Nu=-{\theta }^{^{\prime}}\left(0\right), P{e}^{-\frac{1}{2}}Sh=-{\phi }^{^{\prime}}\left(0\right), P{e}^{-\frac{1}{2}}Nn=-{\chi }^{^{\prime}}\left(0\right).\end{array}$$

## Method of solution

Using similarity transformations, the governing partial differential equations were converted into ordinary differential equations, which are then solved numerically using Matlab bvp4c solver. Matlab bvp4c solver is a finite difference method with fourth-degree accuracy that is applied on a general two-point boundary value problem with an initial solution guess. It does this by integrating a system of ordinary differential equations on the interval $[a, b].$ From this method, and using a diversity of initial guess $f,{f}^{^{\prime}},\theta , {\theta }^{^{\prime}},\phi ,{\phi }^{^{\prime}},\chi ,$ and ${\chi }^{^{\prime}}$ we were able to find the first and second solutions. In the context of the bvp4c function described earlier, we need to transform the governing equations into a system of first order differential equations as follows:

First, we arrange Eqs. (11) through (14) as:

$${f}^{^{\prime\prime} }=\lambda \left[{\theta }^{^{\prime}}-Nr{\phi }^{^{\prime}}-Rb{\chi }^{^{\prime}}\right],$$

$${\theta }^{^{\prime\prime} }=\frac{-\gamma {\theta }^{^{\prime}}-f{\theta }^{^{\prime}}+{f}^{^{\prime}}\theta }{1+\gamma \eta },$$

$${\phi }^{^{\prime\prime} }=\frac{-\gamma {\phi }^{^{\prime}}-Lef{\phi }^{^{\prime}}+Le{f}^{^{\prime}}\phi }{1+\gamma \eta },$$

$${\chi }^{^{\prime\prime} }=\frac{-\gamma {\chi }^{^{\prime}}-Lbf{\chi }^{^{\prime}}+Lb{f}^{^{\prime}}\chi +Pb((1+\gamma \eta ){\phi }^{^{\prime}}{\chi }^{^{\prime}}+(\chi +A)(\gamma {\phi }^{^{\prime}}+(1+\gamma \eta ){\phi }^{^{\prime\prime} }}{1+\gamma \eta }.$$

Next, we transform the above equations into a system of first order differential equations, and for this, we let $\eta =x$, and this gives us

$${y}_{1}=f, {y}_{2}={f}^{^{\prime}},$$

$${y}_{3}=\theta , {y}_{4}={\theta }^{^{\prime}},$$

$${y}_{5}=\phi , {y}_{6}={\phi }^{^{\prime}},$$

$${y}_{7}=\chi , {y}_{8}={\chi }^{^{\prime}}.$$

Therefore, the corresponding system of first order differential equations become:

$$\frac{d{y}_{1}}{dx}={f}^{^{\prime}}={y}_{2,}$$

$$\frac{d{y}_{2}}{dx}={f}^{^{\prime\prime} }=\lambda \left[{y}_{4}-{y}_{6}Nr-{y}_{8}Rb\right],$$

$$\frac{d{y}_{4}}{dx}={\theta }^{^{\prime\prime} }=\frac{-\gamma {y}_{4}-{y}_{1}{y}_{4}+{y}_{2}{y}_{3}}{1+\gamma x},$$

$$\frac{d{y}_{6}}{dx}={\phi }^{^{\prime\prime} }=\frac{-\gamma {y}_{6}-{y}_{1}{y}_{6}Le+{y}_{2}{y}_{5}Le}{1+\gamma x},$$

$$\frac{d{y}_{8}}{dx}={\chi }^{^{\prime\prime} }=\frac{-\gamma {y}_{8}-{y}_{1}{y}_{8}Lb+{y}_{2}{y}_{8}Lb+Pb\left(\left(1+\gamma x\right){y}_{6}{y}_{8}+({y}_{7}+A)(\gamma {y}_{6}+(1+\gamma x)(-\gamma {y}_{6}-Le{y}_{1}{y}_{6}+Le{y}_{2}{y}_{5})\right)}{1+\gamma x}.$$

For the boundary conditions, we consider that $ya$ is the left boundary, and $yb$ be the right boundary such that:

$$ya\left(1\right)=0, yb\left(2\right)-1=0,$$

$$ya\left(3\right)-1=0, yb\left(3\right)=0,$$

$$ya\left(5\right)-1=0, yb\left(5\right)=0,$$

$$ya\left(7\right)-1=0, yb\left(7\right)=0.$$

To validate our results, the differential equations are solved numerically using Maple 14.0 ***dsolve*** command. The asymptotic boundary conditions in Eqs. (15) and (16) are replaced by using a value of $8$ for the similarity variable ${\eta }_{max}=8$. The results for both cases are displayed in Table 1, and they indicate that there are good agreement and preciseness of the numerical calculations. To further validate our results, in Table 2, we compare our present results for the special case against the results of investigations by Chamkha and Khaled^66^ and Nima et al.^67^.

**Table 1:** Effect of curvature parameter $\gamma$ on ${-\theta }^{^{\prime}}\left(0\right)$.

Effect of curvature parameter $\gamma$ on ${-\theta }^{^{\prime}}\left(0\right)$			
$\uplambda$	$\gamma$	${-\theta }^{^{\prime}}\left(0\right)$ (Matlab bvp4c)	${-\theta }^{^{\prime}}\left(0\right)$ (Maple 14.0)
		First solution	First solution
− 1	0.0	0.731408	0.7314073
− 1	0.5	0.873551	0.8735520
− 1	1.0	1.000111	1.0001002
− 1	3.0	1.436533	1.4364811
− 1	5.0	1.824377	1.8243414

**Table 2:** Comparison of ${f}^{^{\prime}}\left(0\right)$ when $\lambda =0$. Here we have ${N}_{1}=0, {N}_{2}=0, m=0, \gamma =0,$ $\omega =0, Lb=0, Le=0, Pe=0, A=0.$

Comparison of ${f}^{^{\prime}}(0)$ for $\lambda =0$			
	Chamkha and Khaled^66^ For $B=0$	Nima et al.^67^ For $\epsilon =1$	Present results (first solution)
${f}^{^{\prime}}(0)$	1.0000	1.0000	1.0000

## Results and discussion

In this section, dual solutions of different flow profiles are analyzed. Dual solutions represent two different branches of solutions that are obtained under the same conditions by guessing some missing initial values. These solutions are called upper branch, or first solution, and lower branch or second solution. In this paper's visualizations, the solid line represents the first solution, while dotted lines represent the second solution. A stability analysis described by Sparrow et al.^68^, Weidman et al.^69^, and very recently Postelnicu and Pop^70^, reveal that upper branch solutions (first solution) are stable and physically realizable. In contrast, lower branch solutions (second solution) are unstable, and therefore not physically realizable.

**Figure 2:**
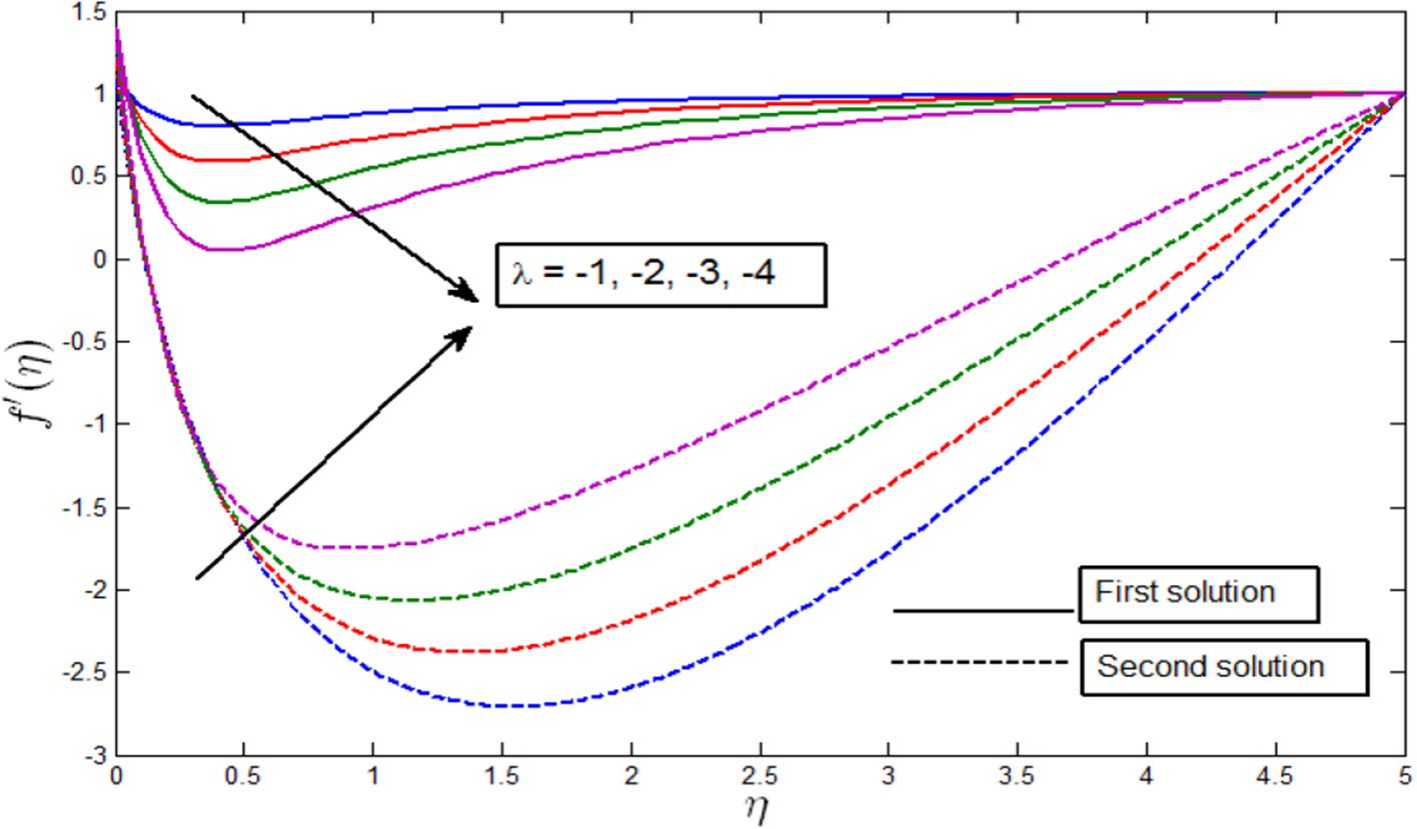
Velocity profile ${f}^{^{\prime}}\left(\eta \right)$ for various $\lambda$ when $\gamma =5, Lb=0.5, Le=0.5, Pb=0.5, Nr=0.5, Rb=0.6, A=0.2.$

Figure 2 shows that multiple solutions are possible for different values of $\lambda .$ For example, when $\lambda <0$, multiple solutions exist. In the same figure, dual solutions are obtained for $\lambda = -1,-2,-3,-4$. Finally, either no solution exists, or a unique solution exists for $\lambda >0$. The figures also show that dual solutions are possible for all values of ${\lambda }_{c}\le \lambda <0$**,** where ${\lambda }_{c}<0$ is the critical value. For the first solution in Fig. 2, the velocity profile increases with the augmented values of mixed convection parameter $\lambda$ for the dominance of buoyancy force for the up-swimming microorganisms.

**Figure 3:**
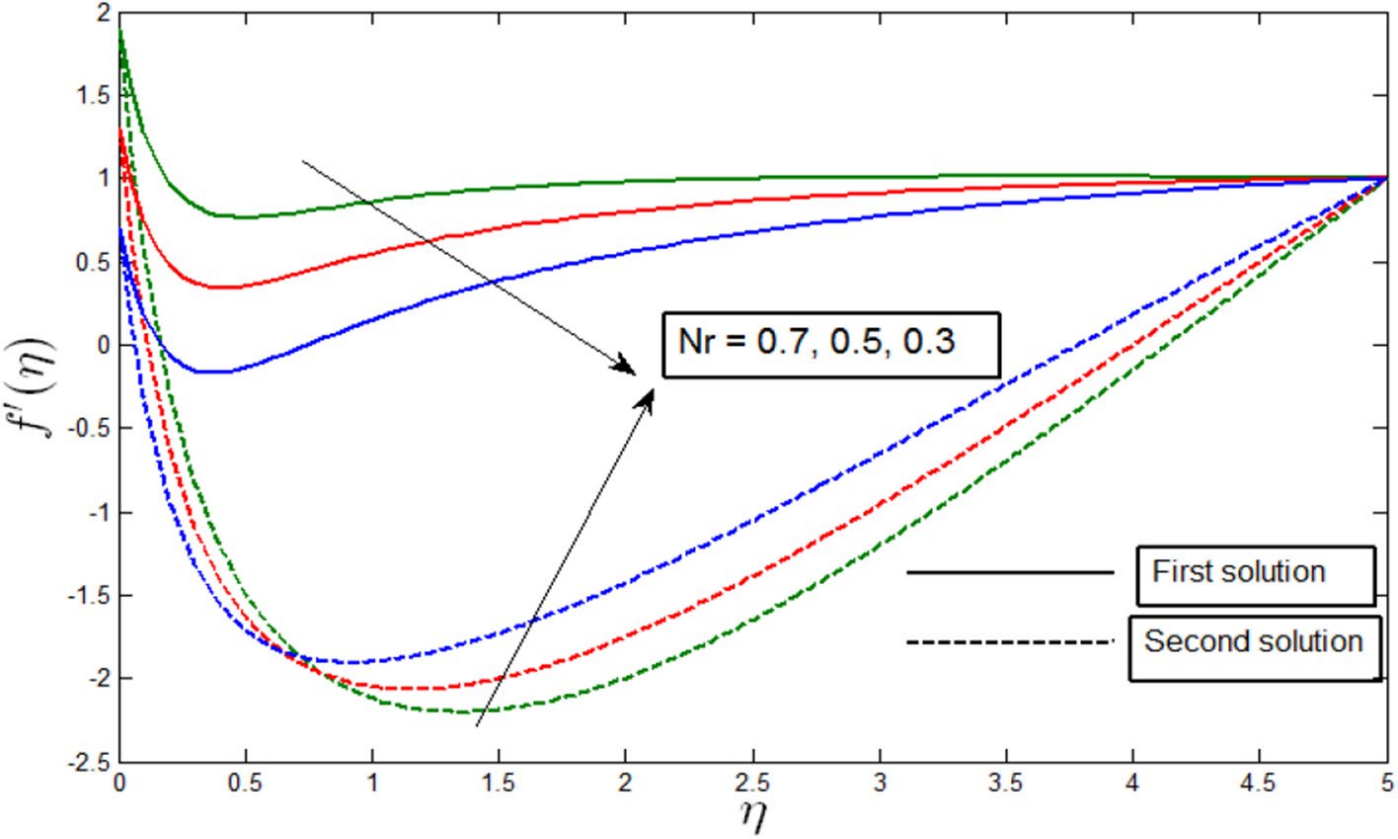
Velocity profile ${f}^{^{\prime}}\left(\eta \right)$ for various $Nr$ when $\lambda =-3, \gamma =5, Lb=0.5, Le=0.5,$ $Pb=0.5, Rb=0.6, A=0.2.$

Figure 3 shows the velocity profile ${f}^{^{\prime}}\left(\eta \right)$ against $\eta$ for random values of $Nr$ when $\lambda =-3$. The velocity profiles provide the existence of the dual solution when $\lambda <{\lambda }_{c}$ with a diversity of $Nr$. From the figure, we see that the first solutions are stable as the velocity profile went into the positive range. We also see that the second solutions are unstable as the velocity profile became negative. Figure 3 illustrates the influence of Buoyancy parameter $Nr$ over the dual solution. Figure 3 shows a decrease in Buoyancy parameter $Nr$, where velocity profile decreases for the first solution but increases in the second solution. Although the second solutions have negative values, there are no physical significances that can be made.

**Figure 4:**
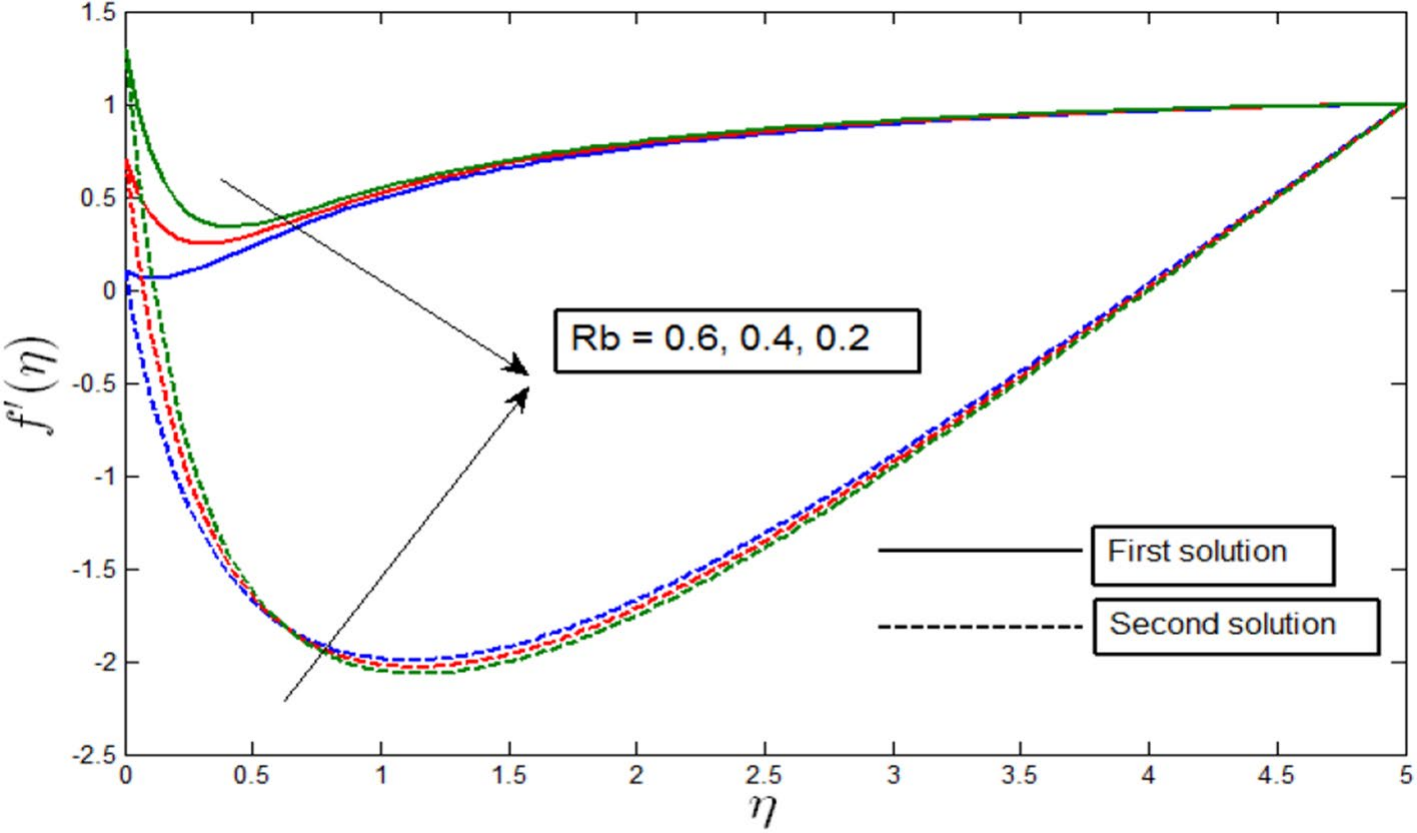
Velocity profile ${f}^{^{\prime}}\left(\eta \right)$ for various $Rb$ when $\lambda =-3, \gamma =5, Lb=0.5, Le=0.5, Pb=0.5, Nr=0.5, A=0.2.$

The velocity profile ${f}^{^{\prime}}\left(\eta \right)$ against $\eta$ for several values of $Rb$ is visualized in Fig. 4 when $\lambda =-3$. The velocity profile provides the existence of the dual solution with $\lambda =-3$ with a certain change of bioconvection Rayleigh number $Rb$. Figure 4 shows the effect of $Rb$ over the dual solution when the curvature parameter $\gamma =5$, Peclet number $Pb = 0.5$, bioconvection Lewis number $Lb=0.5$, Lewis number $Le=0.5$, buoyancy parameter $Nr=0.5$, and microorganism concentration difference parameter $A=0.2$. The greater values of $Rb$ increase the buoyancy force because of the bio-convection process. It is observed in Fig. 4 that when the parameter $Rb \mathrm{is decreased}$, the first solutions of the dual velocity profile decrease, and the second solutions increase, which implies that the first solution is the stable one.

**Figure 5:**
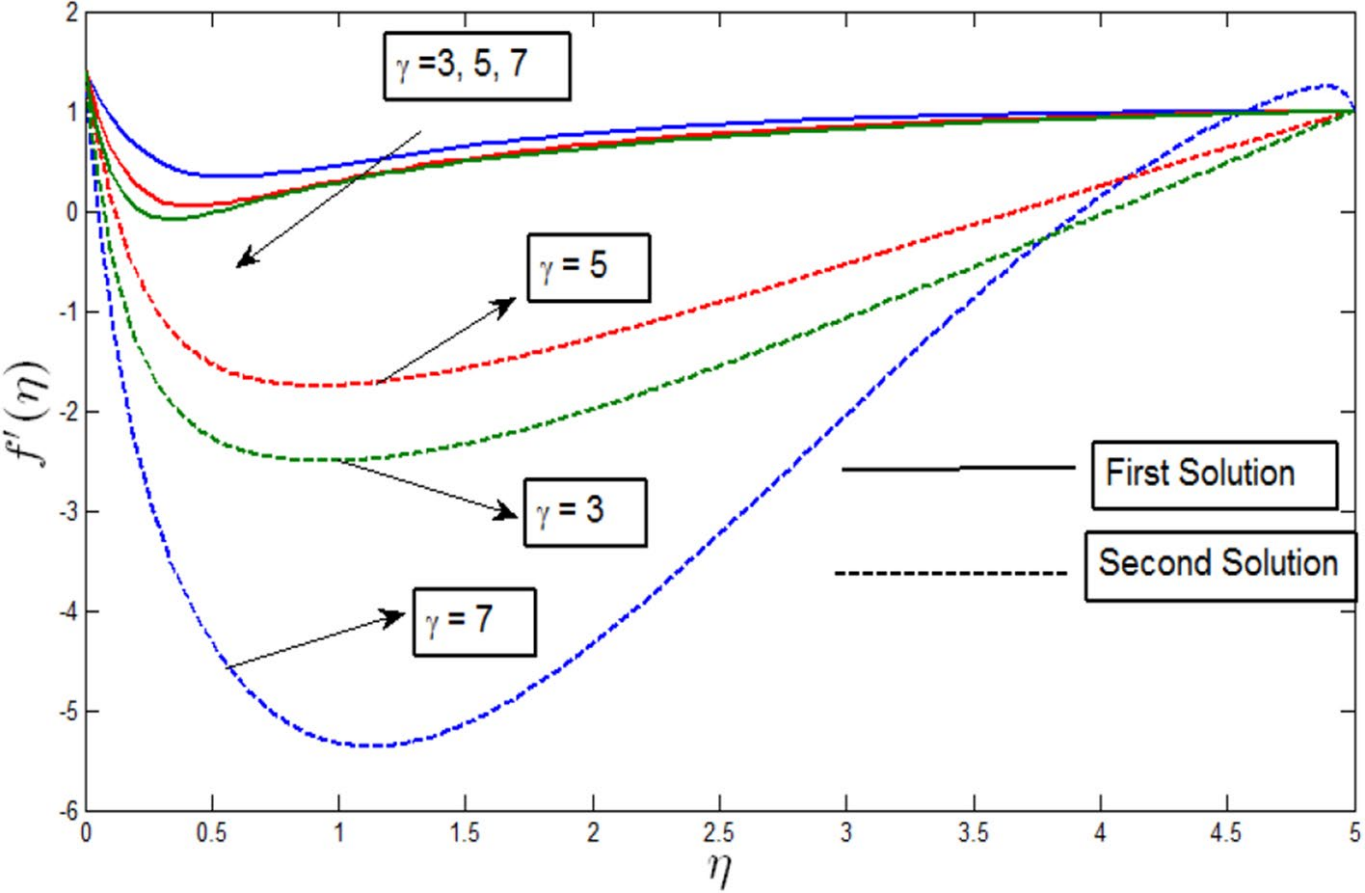
Velocity profile ${f}^{^{\prime}}\left(\eta \right)$ for various $\gamma$ when $\lambda =-4, Lb=0.5, Le=0.5, Pb=0.5, Nr=0.5, Rb=0.6, A=0.2.$

The velocity profile ${f}^{^{\prime}}\left(\eta \right)$ against $\eta$ for several values of $\gamma$ is shown in Fig. 5 for $\lambda =-4$. The velocity profiles provide the existence of the dual solution with $\lambda =-4$ with a certain change of curvature parameter $\gamma$. Figure 5 shows the effect of $\gamma$ over the dual solution when the bioconvection Rayleigh number $Rb=0.6$, Peclet number $Pb = 0.5$, bioconvection Lewis number $Lb=0.5$, Lewis number $Le=0.5$, Buoyancy parameter $Nr=0.5,$ and the microorganism concentration difference parameter $A=0.2$. The curvature parameter has an inverse relation with the radius of curvature. Thus, when the curvature parameter increases, the radius of the cylinder decreases. Also, less contact within the surface area will produce less resistance towards the fluid particles. As a result, the velocity profile shows stimulant values.

**Figure 6:**
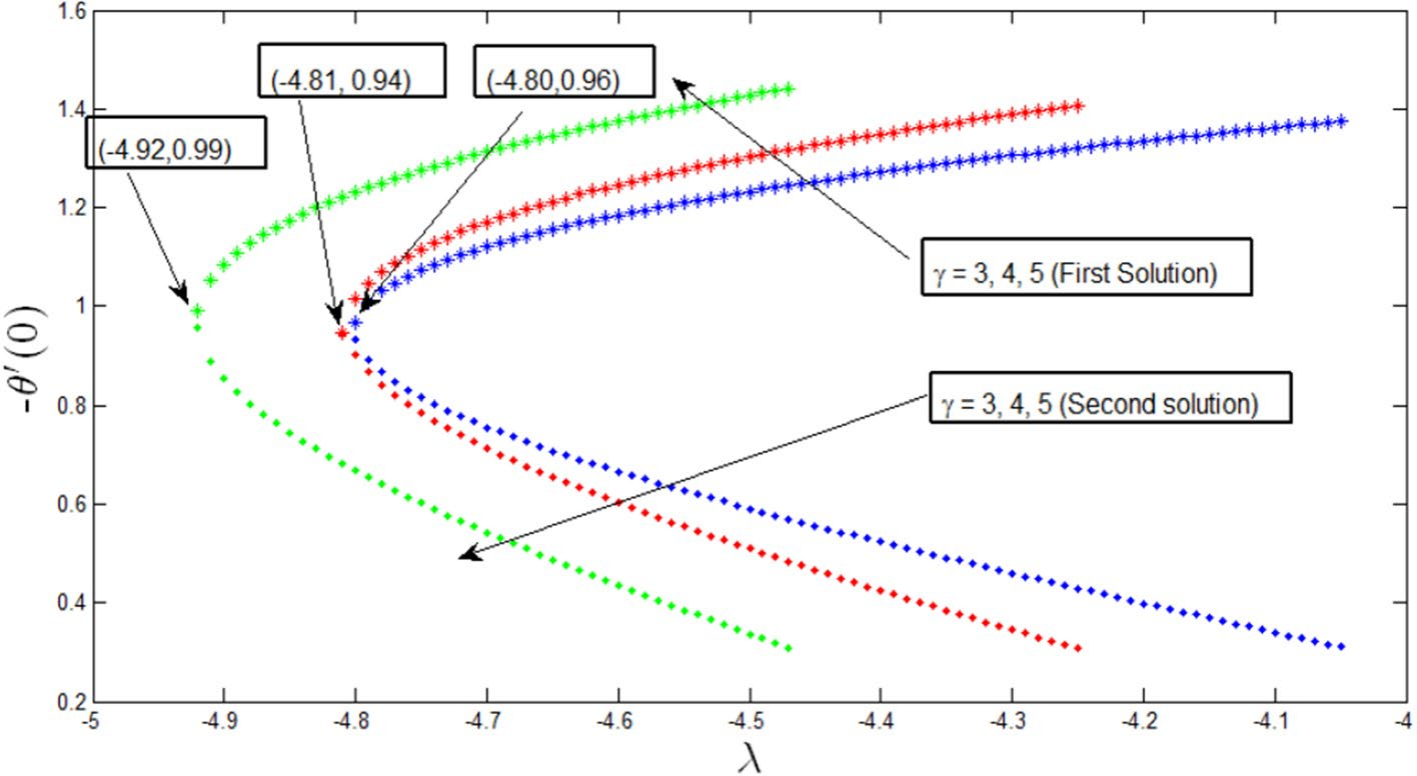
Variation of Nusselt number with $\lambda$ for various $\gamma$ when $Lb=1, Le=1, Pb=1,$ $Nr= 0.5, Rb=0.6, A=0.2.$

Variation of Nusselt number with $\lambda$ for different values of $\gamma$ is shown in Fig. 6. It is seen that dual solutions exist for the temperature profile $\lambda >{\lambda }_{c}$ where ${\lambda }_{c}=-4.80, -4.81,-4.92,$ and $\gamma =3, 4, 5,$ respectively. The critical value ${\lambda }_{c}$ is where both the upper and lower branch solutions connect, and at this exact point, a unique solution exists. From these critical values, the boundary layer separates, and the solution becomes invalid. It is found from the heat transfer rate $-{\theta }^{^{\prime}}\left(0\right)$ that it increases strongly with the parameter $\uplambda$ and decreases relatively weakly with the curvature parameter $\gamma$.

**Figure 7:**
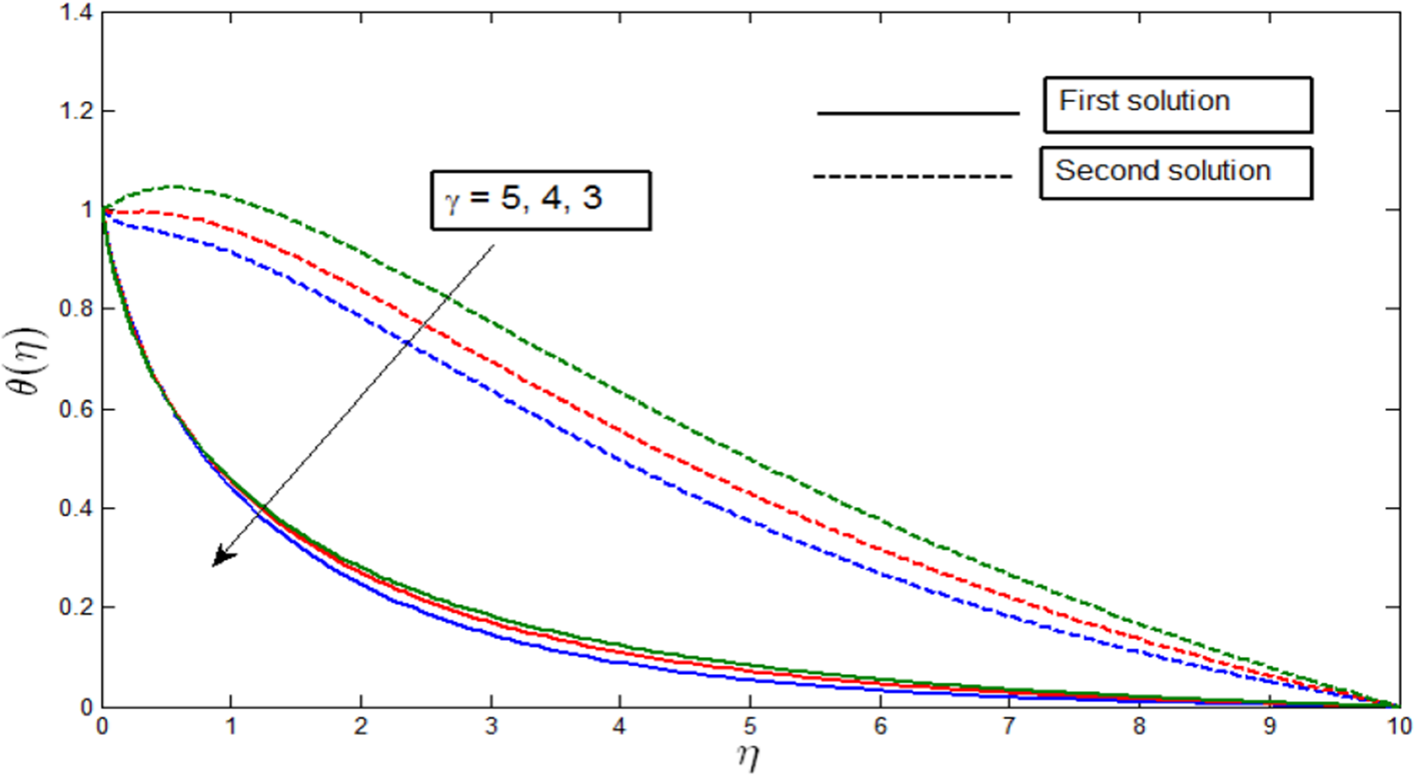
Temperature profile $\theta \left(\eta \right)$ for various $\gamma$ when $\lambda =-4, Lb=1, Le=1, Pb=1, Nr=0.5, Rb=0.6, A=0.2.$

Figure 7 shows the temperature profile $\theta \left(\eta \right)$ against $\eta$ for different values of $\gamma$ when $\lambda =-4$. The temperature profiles provide the existence of the dual solution when $\lambda >{\lambda }_{c}$ for different values of $\gamma$. An increase of the curvature parameter $\gamma$ causes a decrease in curvature radius because the fluid velocity particle enhances. As a result, the average kinetic energy increases, which causes an increment in the temperature profile. It is seen in Fig. 7 that when the curvature parameter $\gamma$ decreases, the temperature profiles also decrease for both solutions.

**Figure 8:**
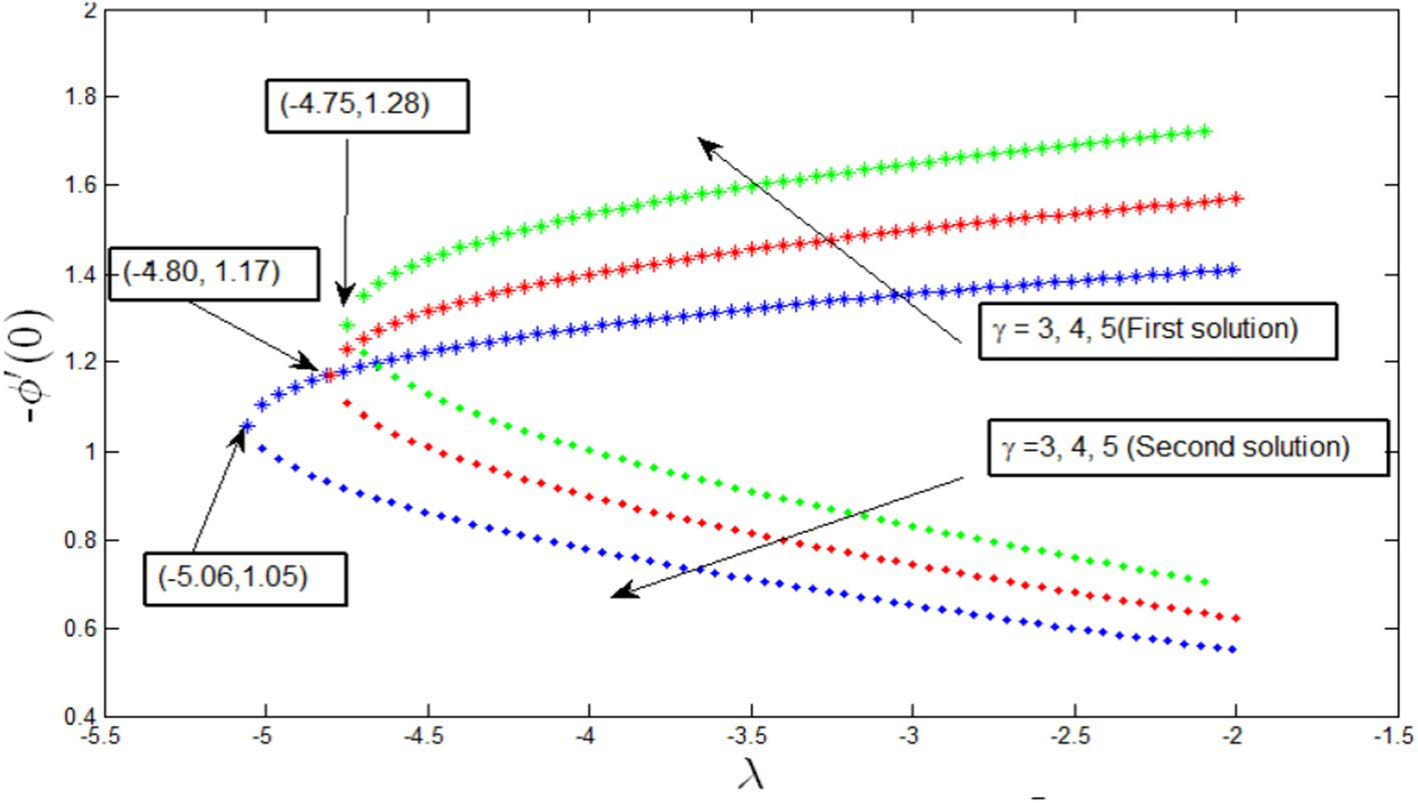
Variation of Sherwood number with $\lambda$ for various $\gamma$ when $Lb=1, Le=0.5, Pb=1, Nr=0.5, Rb=0.6, A=0.2.$

Variation of Sherwood number with $\lambda$ for different values of $\gamma$ is shown in Fig. 8. The dual solution is observed for the concentration profile $\lambda >{\lambda }_{c}$, where ${\lambda }_{c}=-4.75, -4.80,-5.06,$ and $\gamma =3, 4, 5,$ respectively. At this critical point ${\lambda }_{c}$, a unique solution exists. Under these critical values, the boundary layer separates, and the solution-based become invalid. It is also observed that the Sherwood number increases with the increasing values of $\lambda$ and $\gamma$ for the first solution, while it decreases for the second solution.

**Figure 9:**
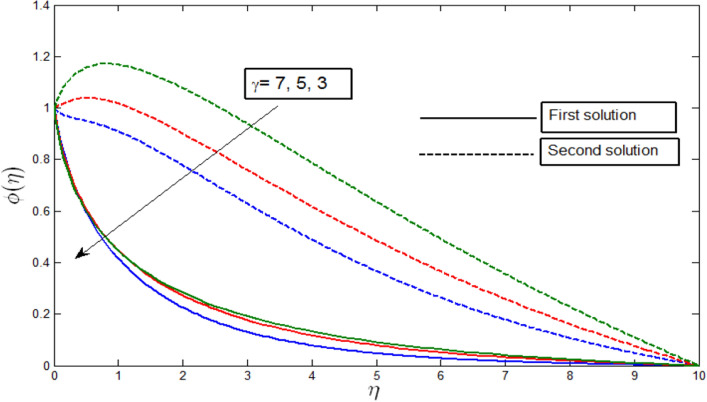
Concentration profile $\phi \left(\eta \right)$ for various $\gamma$ when $\lambda =-4, Lb=0.5, Le=1, Pb=0.5, Nr=0.5, Rb=0.6, A=0.2.$

Figure 9 shows the concentration profile $\phi \left(\eta \right)$ against $\eta$ for random values of $\gamma$ when $\lambda =-4$. The concentration profile provides the existence of the dual solution when $\lambda >{\lambda }_{c}$ with different values of $\gamma$. We see that both solutions are stable as the velocity profile went into the positive range. Figure 9 illustrates the influence of curvature parameter $\gamma$ over the dual solution. We also see that a decrease in curvature parameter $\gamma$ makes the concentration profile decrease for both the first and second solutions.

**Figure 10:**
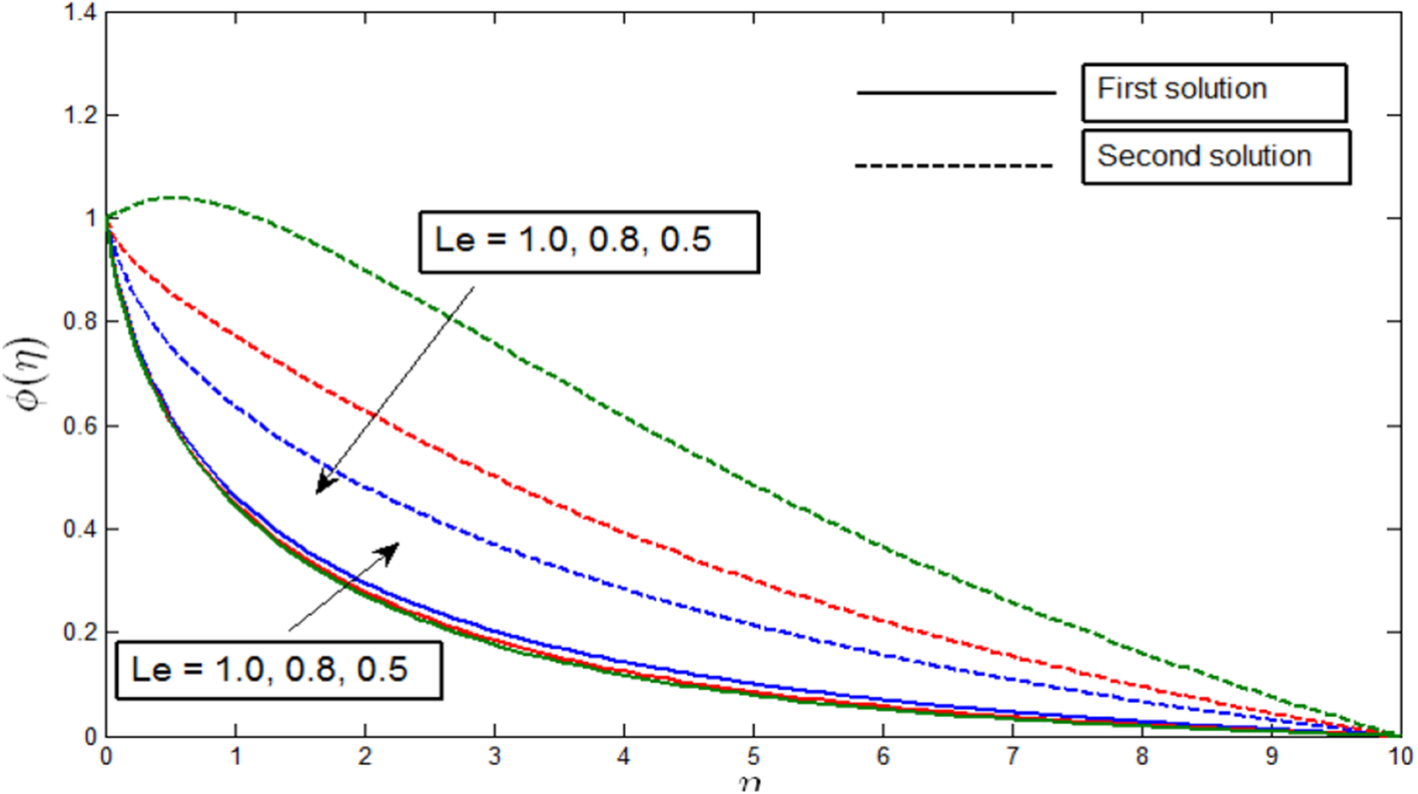
Concentration profile $\phi \left(\eta \right)$ for various $Le$ when $\lambda =-4, \gamma =5, Lb=0.5, Pb=0.5, Nr=0.5, Rb=0.6, A=0.2.$

The concentration profile $\phi \left(\eta \right)$ against $\eta$ for several values of $Le$ in Fig. 10 when $\lambda =-4$. The concentration profiles provide the existence of the dual solution with $\lambda =-4$ ($\lambda >{\lambda }_{c}$) with a certain change of Lewis number $Le$. Both solutions are shown to be stable as the concentration profile went into the positive range. Figure 10 shows the effect of $Le$ over the dual solution when the curvature parameter $\gamma =5$, Peclet number $Pb = 0.5$, bioconvection Lewis number $Lb=0.5$, bioconvection Rayleigh number $Rb=0.6$, buoyancy parameter $Nr=0.5$, and the microorganism concentration difference parameter $A=0.2$. The Lewis number $Le$ is defined as the ratio of thermal diffusivity and mass diffusivity, which is the prominent factor in studying heat and mass transfer. As Lewis number $Le$ reduces the mass diffusivity, this in turn decreases the penetration depth of the concentration boundary layer. We observe in Fig. 10 that as the parameter $Le$ decreases, the first solutions of concentration profile increase, and the second solutions decrease.

**Figure 11:**
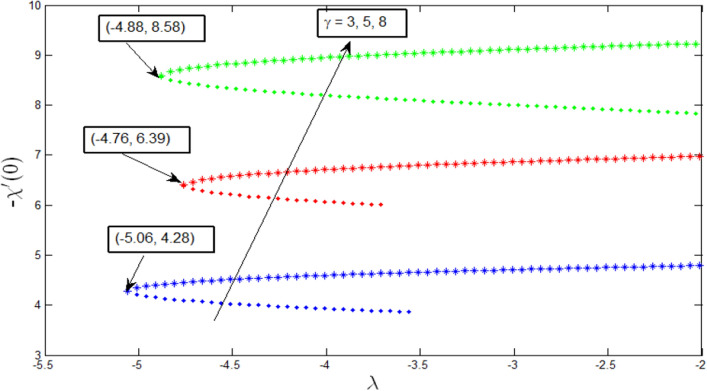
Microorganism transfer rate $-{\chi }^{^{\prime}}\left(0\right)$ with $\lambda$ for various values of $\gamma$ when $Pb=0.5, Le=1, Nr=0.5, Rb=0.6, A=0.2.$

In Fig. 11, the density of motile microorganism transfer rates is also increased with the mixed convection parameter and curvature parameter. It is known that the motile microorganism density is higher than liquid, and they usually swim in an upward direction of the exterior of the cylinder wall. Therefore, the curvature parameter $\gamma$ increases the motile microorganism transfer rate. The dual solution is observed for the microorganism profile $\lambda >{\lambda }_{c}$, where ${\lambda }_{c}=-5.06, -4.76, -4.88,$ and $\gamma =3, 5, 8,$ respectively. At this critical point ${\lambda }_{c}$, a unique solution exists. From these critical values, the boundary layer separates, and the solution is based on becomes invalid. It is found from microorganism transfer rate $-{\chi }^{^{\prime}}\left(0\right)$ that it increases strongly with parameter $\uplambda$, and grows relatively stronger with curvature parameter $\gamma$.

**Figure 12:**
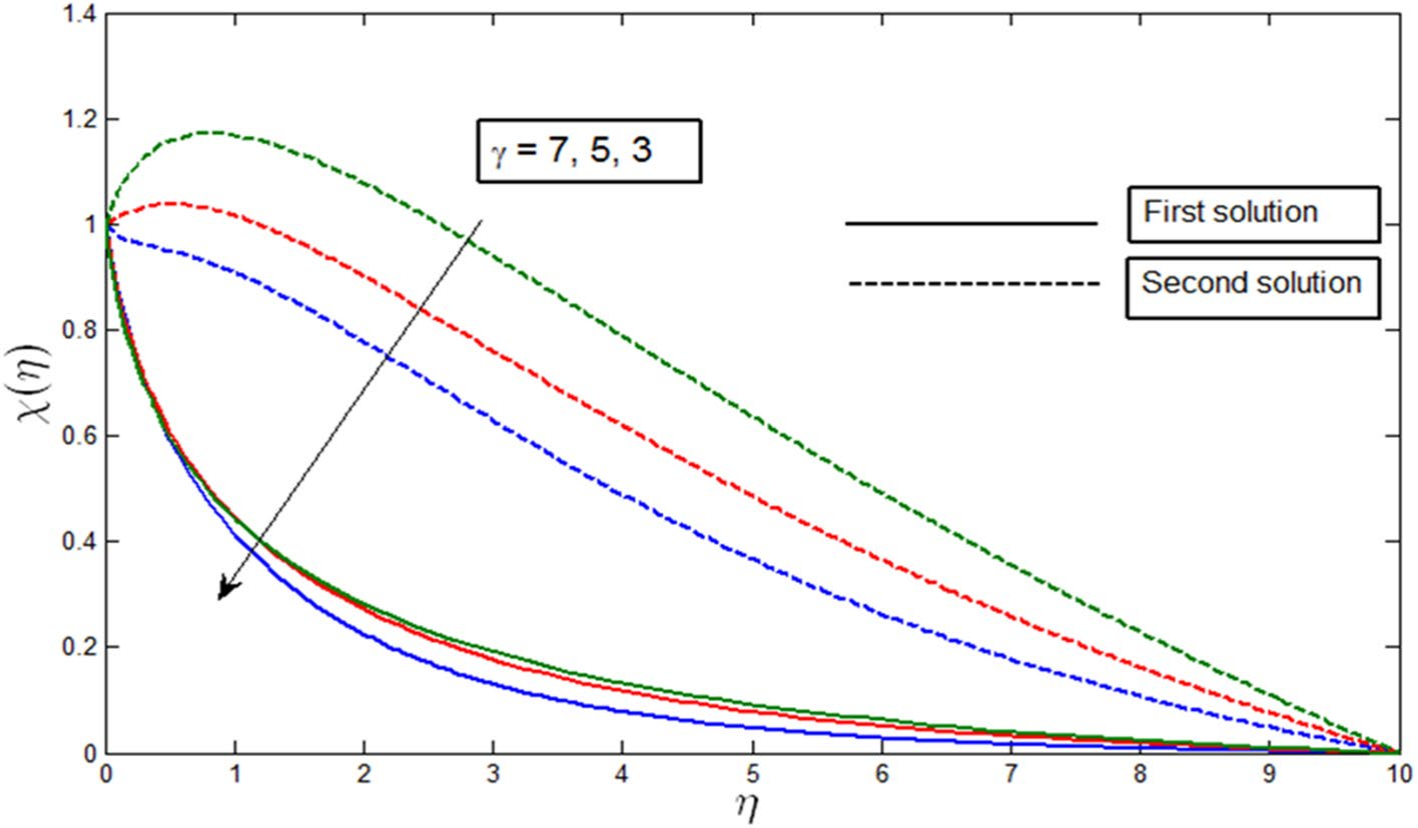
Microorganism profile $\chi \left(\eta \right)$ for various $\gamma$ when $\lambda =-4, Lb=0.5, Le=1, Pb=0.5, Nr=0.5, Rb=0.6, A=0.2.$

**Figure 13:**
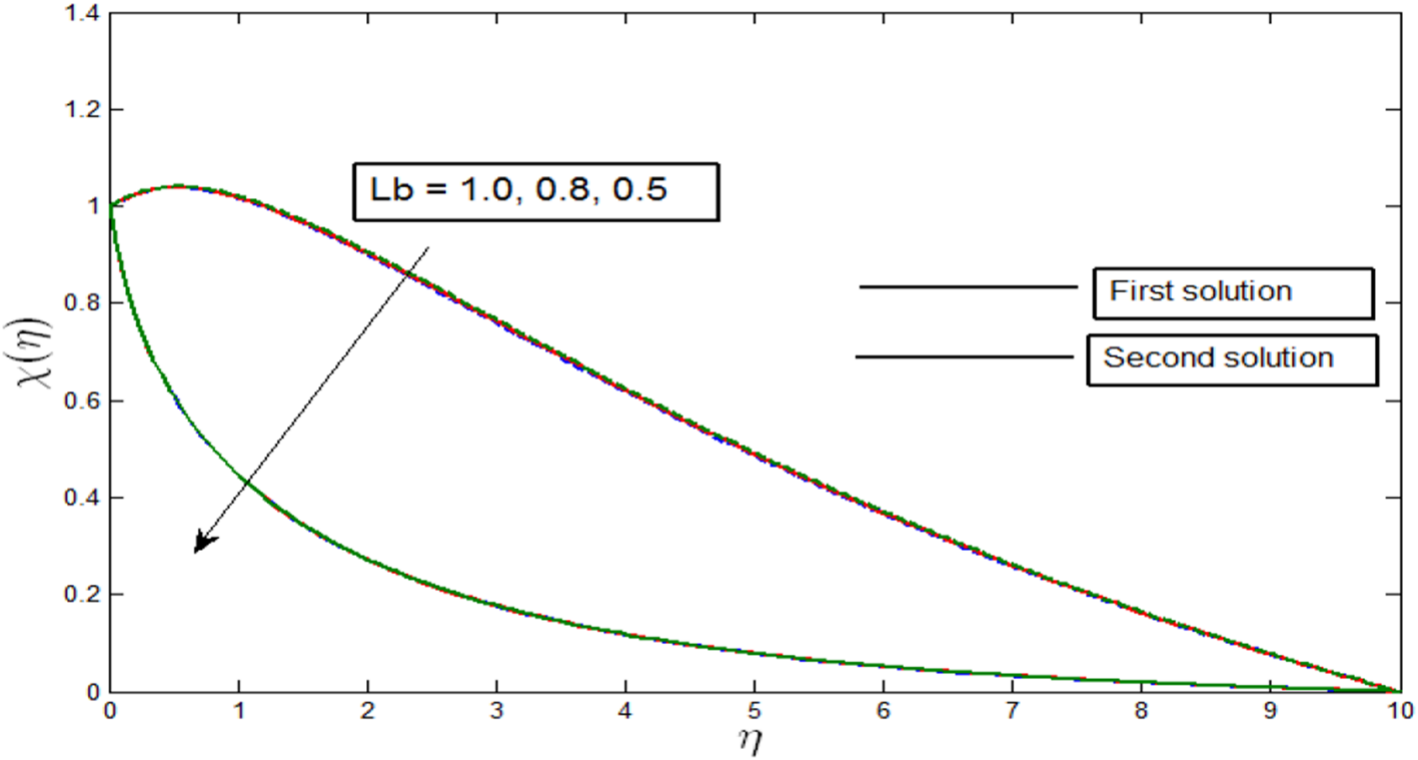
Microorganism profile $\chi \left(\eta \right)$ for various $Lb$ when $\lambda =-4, \gamma =5, Le=1, Pb=0.5, Nr=0.5, Rb=0.6, A=0.2.$

**Figure 14:**
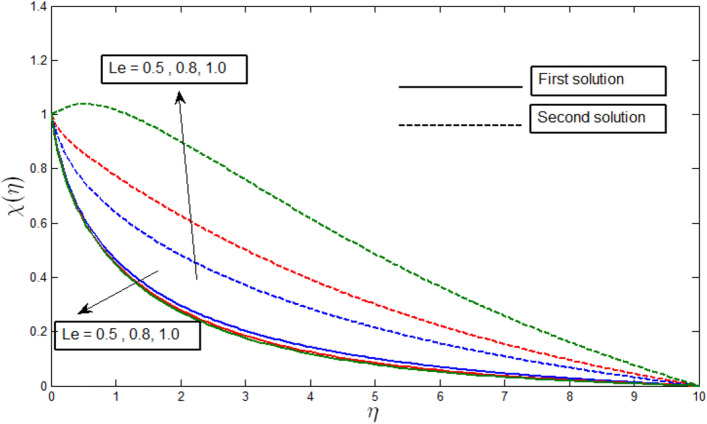
Microorganism profile $\chi \left(\eta \right)$ for various $Le$ when $\lambda =-4, \gamma =5, Le=1, Pb=0.5, Nr=0.5, Rb=0.6, A=0.2.$

**Figure 15:**
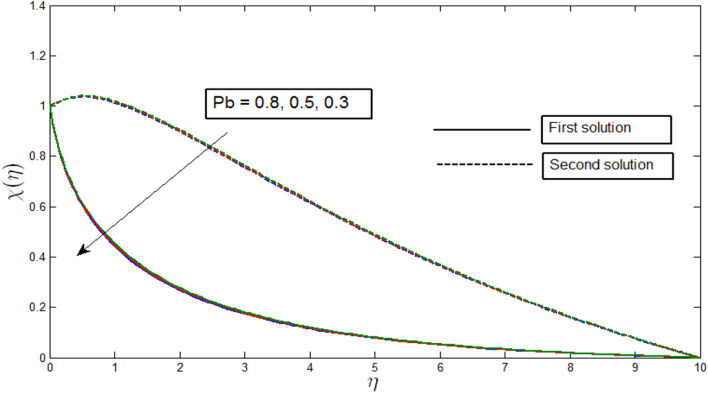
Microorganism profile $\chi \left(\eta \right)$ for various $Pb$ when $\lambda =-4, \gamma =5, Le=1, Nr=0.5, Rb=0.6, A=0.2.$

Figures 12, 13, 14 and 15 show the existence of dual solution of microorganism profile when $\lambda >{\lambda }_{c}$ for the values $\gamma =3, 5, 7, Le = 0.5, 0.8, 1.0, Lb = 0.5, 0.8, 0.1, \mathrm{and} Pb = 0.3, 0.5, 0.8$ is shown. The Bioconvection Lewis number $Lb$ and bioconvection Peclet number $Pb$ tend to decrease the microorganism profile. In addition, the Bioconvection Lewis number $Lb$ and bioconvection Peclet number $Pb$ raises the mobility of fluid and causes the quantity of motile microorganism’s thickness to reduce. Microorganism profiles decrease with the decreasing values of $\gamma , Lb,$ and $Pb$ for both the first and second solutions. Additionally, increasing the Lewis number’s values also decreases the boundary layer thickness of the microorganism profile for the first solution, where the profile increases with $Le$ in the second solution. We see that both solutions are stable as the microorganism profile went into the positive range.

## Conclusion

The steady mixed convection boundary layer flow with gyrotactic microorganisms past a vertical cylinder is analyzed. Dual solutions are found to exist in case of opposing flow when the mixed convection parameter $\lambda$ is negative (Cylinder is cooled ${T}_{w}<{T}_{\infty })$. The consequences of various flow influencing parameters have been thoroughly discussed in detail. The critical reviews are summarized as follows:

- The variation of Nusselt number indicates that dual solutions exist for temperature profile $\uplambda >{\lambda }_{c},$ where the critical value ${\lambda }_{c}=-4.80, -4.81,-4.92$ for the curvature parameter $\gamma =3, 4, 5$. The curvature parameter γ increases heat transfer rate and temperature profile for the first solution, which is physically stable.
- The variation of Sherwood number shows the existence of dual solutions in concentration profile when $\lambda >{\lambda }_{c}=-4.75, -4.80, -5.06$ for $\gamma =3, 4, 5,$ respectively. The mass transfer rate and concentration profile increase due to the dependence on curvature parameter $\gamma$ and Lewis parameter $Le$ for the stable solutions.
- The variations of density number of microorganisms show the dual solution of microorganism profile arise when $\lambda >{\lambda }_{c}=-5.06, -4.76,-4.88$ for $\gamma =3, 5, 8,$ respectively. For the case of stable solutions, motile microorganism transfer rate and microorganism profile increase with the enhancement of $\gamma ,$ and it is observed that the bioconvection Lewis parameter $Lb$ and Bioconvection Peclet number $Pb$ have pronounced effects on Microorganism profile.

Several studies were performed on dual solutions for mixed convection along a vertical cylinder for different engineering applications. Moreover, there are many engineering and practical bio-microsystems where mixed convection flow over a vertical cylinder in porous media with Gyrotactic Microorganism occurs. However, very few works have been done on dual solutions for mixed convection with gyrotactic microorganisms. Analyzing the existence of a dual solution in heat, mass, motile microorganism transfer rate, temperature, and concentration microorganism profile beyond a critical point along a vertical cylinder is a novel concept. The obtained results are also unique. Our study shows mutual relations between different parameters, which can affect the performance of those systems.

In this paper, dual solution phenomena in the presence of gyrotactic microorganisms are observed only in the case of mixed convective opposing flow. For further extensions of this paper, we can consider non-Newtonian fluid with the effect of an aligned magnetic field to observe dual solution phenomena for both assisting flow and opposing flow.
